# A Gas Leakage Localization Method Based on a Virtual Ultrasonic Sensor Array

**DOI:** 10.3390/s19143152

**Published:** 2019-07-17

**Authors:** Lei Li, Kuan Yang, Xiaoyu Bian, Qinghui Liu, Yizhuo Yang, Fengying Ma

**Affiliations:** 1 School of Physics and Engineering, Zhengzhou University, Zhengzhou 450001, China; 2 Glasgow College, University of Electronic Science and Technology of China, Chengdu 610000, China

**Keywords:** gas leakage, localization, virtual phased array, ultrasonic sensor

## Abstract

In traditional sensory array-based acoustic emission methods that are used for gas leakage localization, the localization resolution depends on the spatial aperture of the array, that is, the number of sensors. Most of the existing methods use small arrays that can only achieve low-resolution localization results because of limitations such as the amplitude and phase consistency, the complexity and cost of the system. This paper reports the first application of a virtual phased array for gas leakage detection to obtain high-resolution localization results. This method uses a virtual linear ultrasonic sensor array composed of only two sensors to acquire leakage signals. Then, we use the virtual beamforming algorithm based on the cross-power spectrum to estimate the location of the leakage source. Several experiments were conducted to evaluate the effectiveness and operability of the proposed method. The impacts of various factors on the performance of the localization technique are compared and discussed, including factors such as the number of sensors and the distance between the leak hole and virtual array. The results demonstrate that the proposed method accurately and reliably localizes gas leakages.

## 1. Introduction

Gas leakage, which leads to serious environmental damage and industrial hazards, is of great importance in the industrial field, with leakage sources including pressure vessels, pipes and tanks [1,2,3]. Therefore, establishing a method for the quick detection and accurate localization of leakage sources has become a challenge that demands an urgent solution. Currently, common approaches to leakage detection and localization include mass/volume balance, negative pressure waves, distributed optical fibers, the resistance change method and the acoustic emission (AE) method [4]. The AE method, a nondestructive testing technique, is used to estimate the location of leakage holes by analyzing the acoustic signal of the leakage. Compared with other methods, the AE method is superior—it has higher sensitivity, higher location accuracy, a lower false alarm rate, a shorter testing time and greater adaptability [5,6,7].

Two main types of AE methods can be defined: a detection method with a time domain and a localization method with a spatial domain. The time-domain detection method can be used to calculate and verify the leakage location by using the acoustic propagation velocity and the time difference in the arrival of an acoustic signal at the two ends of a pipeline [8,9]. With this approach, the leakage can be detected using a time–frequency technique [10] or a low-frequency impulse method [11]. Li et al. [12] located leakages in gas pipelines using the cross-time–frequency spectrum of leakage-induced acoustic vibrations. To further improve the applicability of the AE technique for a long-range pipeline, a leakage localization approach based on a multi-level framework was proposed in 2016; it includes two steps, namely, regional localization and precise localization [13]. In 2019, Thang et al. [14] proposed a reliable acoustic emission-based technique for the detection of a small leakage in a pipeline system and it obtained results with better quality and more reliability compared with other methods. In the same year, a method for the localization of a CO${}_{2}$ gas leakage by emission multi-sensor fusion based on wavelet-radial basis function network (RBFN) modeling was proposed [15]. Although these methods have the advantage of high detection accuracy, their stability performance is poor because the localization accuracy of time-domain methods is susceptible to the effects of various factors, such as ambient temperature, gas flow rate and gas pressure. The other main type of AE method, the spatial-domain method, uses an ultrasonic sensor array at a certain distance from the pipeline to record the ultrasonic signal generated by the leakage. Then, it uses spatial signal processing technology, such as beamforming technology, to locate the leakage source [16,17,18]. A time–space domain correlation-based method was proposed in which the propagated signal is acquired on the skin of the plate by using an L-type array of 8 elements [19]. Yan et al. [20] presented the principle and application of a linear acoustic emission sensor array of 6 elements and a near-field beamforming technique to identify the location of a continuous CO${}_{2}$ leakage. On the basis of previous research, a gas leakage location detection method was proposed in 2018 that applies data fusion of the time difference in signal arrival and energy decay using a planar ultrasonic sensor array of 4 elements [21]. In the same year, a new method for the localization of multiple leakage sources was proposed; it uses a linear array of 4 elements as well as the MUSIC algorithm and wavelet packet analysis [22]. These methods have further improved the accuracy of locating leakage compared with time-domain methods. However, their localization resolution is limited because of insufficient spatial sampling due to too few sensors. There are multiple reasons for using a small number of array elements in these methods. First, integrating a large number of sensors into the array increases the complexity and cost of the system. Secondly, if the array consists of too many sensors, the amplitude and phase consistency of several receiving channels is biased [23].

In order to obtain high-resolution gas leakage localization results, this paper proposes a method for the localization of a leakage source using a virtual phased array (VPA) of only two sensors. The idea of creating a synthetic array or VPA with a limited number of sensors has been explored in many disciplines, with early applications used in radar [24] and sonar systems [25]. Now, it has been widely adopted in many fields, including Wi-Fi holography [26], acoustic field reconstruction [27] and medical tomography [28]. However, in the field of gas leakage detection, the VPA has not been implemented after the investigation stage. In this study, a VPA was applied for the first time in the field of gas leakage detection and it eliminates many practical constraints imposed by conventional arrays. In this method, the amplitude information of the sound field data is recorded by sequentially scanning with one sensor and the phase information is obtained by using a reference sensor. The virtual beamforming algorithm based on the cross-power spectrum is used to estimate the location of the leakage source. This paper presents the theoretical basis of a gas leakage detection method based on a VPA. In addition, experiments were conducted to evaluate the effectiveness, operability and errors analysis of the proposed method.

## 2. Theory

### 2.1. Signal Model

When a gas leakage occurs in gas pipes, the gas flow at the leakage hole is considered isentropic. Ma proposed that the main factors influencing the sound pressure are the nozzle diameter and the ratio of internal pressure to external pressure [29]. The noise level equation can be expressed as

(1) $$L=80+20\mathrm{lg}\frac{D}{D_{0}}+10\mathrm{lg}\frac{{\left(P-P_{0}\right)}^{4}}{P_{0}^{2}{\left(P-0.5P_{0}\right)}^{2}},$$

where *L* is the sound pressure level at a distance of 1 m; *P* is the internal pressure (kPa); $P_{0}$ is the ambient atmospheric absolute pressure (kPa); *D* is the nozzle diameter (mm); and $D_{0}$ is the reference diameter (1 mm).

The ultrasonic signal of a leakage propagates through air with attenuation effects, which primarily include absorption attenuation, scattering attenuation and diffusion attenuation. When an ultrasonic wave propagates through a medium, its energy gradually decays with increasing distance. The attenuation equation is

(2) $$P=P_{s}ue^{j(2\pi \omega t+\varphi )},$$

where $P_{s}$ is the sound pressure in the outlet of the leakage; *u* denotes the propagation operator of amplitude attenuation; ω is the frequency of the ultrasonic signal; φ is the initial phase of the leakage ultrasonic signal; and *t* is the time required for the signal to propagate from its source to the sensor and affects the phase of the received signal.

In this study, a virtual phased array was used to receive the leakage signal. For a conventional array of *M* elements, the signal received by the *i*th array element can be expressed as

(3) $$x_{i}^{*}\left(t\right)=P_{s}u_{i}e^{j(2\pi \omega t_{i}+\varphi )}\;i=1,2,\cdots ,M.$$

For a virtual array of *M* elements, the signal received by the *i*th array element can be expressed as

(4) $$x_{i}\left(t\right)=P_{s}u_{i}e^{j\{2\pi \omega \left[t_{i}+\left(i-1\right)\Delta t\right]+\varphi \}}\;i=1,2,\cdots ,M,$$

where Δt is time difference due to the scanning interval.

A comparison between Equations (3) and (4) reveals that the signal received by the virtual phased array has a time difference Δt due to the scanning interval compared with the signal received by the conventional array. To use conventional array signal-processing techniques for estimating the direction of the leakage source, we must first eliminate this time difference Δt.

### 2.2. Signal Preprocessing

In the method proposed in this paper, the cross-power spectrum is calculated to eliminate the time difference Δt and a reference sensor works simultaneously with the scanning sensor. The signal received by the reference sensor can be expressed as

(5) $$x_{ref}\left(t\right)=P_{s}u_{ref}e^{j\{2\pi \omega \left[t_{r}+\left(i-1\right)\Delta t\right]+\varphi \}}\;i=1,2,\cdots ,M.$$

$F_{x_{i}}\left(f\right)$ and $F_{x_{ref}}\left(f\right)$ are the Fourier transforms of $x_{i}\left(t\right)$ and $x_{ref}\left(t\right)$, respectively. Then,

(6) $$\left\{\begin{array}{c} F_{x_{i}}\left(f\right)=\int_{-\infty }^{\infty }x_{i}\left(t\right)e^{-j2\pi ft}dt,\\ F_{x_{ref}}\left(f\right)=\int_{-\infty }^{\infty }x_{ref}\left(t\right)e^{-j2\pi ft}dt, \end{array}\right.$$

(7) $$F_{x_{ref}}\left(f\right)=e^{-j2\pi f(t_{r}-t_{i})}F_{x_{i}}\left(f\right).$$

The cross-power spectrum of signals $x_{i}\left(t\right)$ and $x_{ref}\left(t\right)$ is denoted by Z(f), which is calculated as

(8) $$Z\left(f\right)=F_{x_{i}}\left(f\right){F_{x_{ref}}}^{*}\left(f\right)={\left|F_{x_{i}}\left(f\right)\right|}^{2}e^{j2\pi f(t_{r}-t_{i})},$$

where ${F_{x_{ref}}}^{*}\left(f\right)$ is the conjugate frequency spectrum of signal $x_{ref}\left(t\right)$. According to Equation (8), the cross-power spectrum is only related to the frequency *f* of the signal and the time difference $t_{r}-t_{i}$ of the two signals. In the following, $\varphi_{i}$ is the phase difference between the *i*th virtual element and the reference sensor.

(9) φi=2πf(tr−ti)=arctanIm[Z(f)]Re[Z(f)].

Our method aims to obtain the phase difference between each virtual array element’s signal by using the cross-power spectrum between each array element’s signal and the reference signal. Then, $\varphi_{i}$ and the standard signal $x_{1}$ can be used to reconstruct the array signal for subsequent localization calculations. The signal of each virtual array element can be expressed as

(10) $$x_{i}=x_{1}e^{2j\pi (\varphi_{i}-\varphi_{1})}\;i=1,2,\cdots ,M.$$

In the above method, the scanning interval Δt is eliminated from the reconstructed signal $x_{i}$. Although $t_{r}$ is superfluous in the reconstructed signal, it is a constant and does not affect the calculation results.

### 2.3. Principle of the Location Algorithm

After the signal received by the virtual array is preprocessed, the time difference due to the scanning interval is eliminated. Then, the beamforming algorithm is used to estimate the direction of the leakage source.

If the incident direction of the plane wave propagating toward the array is α, then

(11) $$\alpha ={\left[sin\theta cos\phi \;sin\theta sin\phi \;cos\phi \right]}^{T},$$

where θ is the azimuth angle and ϕ is the pitch angle.

The direction of the arrival wave α and the position coordinates of each array element $p_{i}$ are used to obtain the time difference caused by the positions of the array elements relative to the received signals. The time difference $\tau_{i}$ is

(12) $$\tau_{i}=\alpha p_{i}^{T}/c,$$

where *c* is the acoustic wave velocity in the medium and c=344 m/s.

For plane waves propagating through a locally uniform medium, the wave number k is defined by

(13) $$k=\frac{\omega }{c}\alpha =\frac{2\pi }{\lambda }\alpha ,$$

where ω is the frequency of the signal and λ is the signal wavelength.

Next, we define

(14) $$V_{k}\left(k\right)={\left[\begin{array}{cccc} e^{-jk^{T}p_{1}} & e^{-jk^{T}p_{2}} & \cdots & e^{-jk^{T}p_{M}} \end{array}\right]}^{T},$$

where $V_{k}\left(k\right)$ is the array manifold vector of the virtual array and it contains all spatial features of the virtual array.

The matrix of the virtual array signals is

(15) $$X\left(\omega \right)={\left[\begin{array}{cccc} x_{1}\left(\omega \right) & x_{2}\left(\omega \right) & \cdots & x_{M}\left(\omega \right) \end{array}\right]}^{T},$$

Then, we can use the received signal and the array manifold vector to calculate the output of the beamforming algorithm. The output of the beamforming algorithm is

(16) $$Y\left(\omega ,k\right)=V_{k}^{H}\left(\tau \right)X\left(\omega \right),$$

where *H* is the conjugate transpose. The output power is

(17) $$P={E[|Y\left(\omega ,k\right)|}^{2}]=V_{k}^{H}\left(\tau \right)RV_{k}\left(\tau \right)$$

where R is the covariance matrix of X(t).

(18) $$R=E\left[X\left(\omega \right)X^{H}\left(\omega \right)\right].$$

The principle of the beamforming algorithm is to search for the direction of the largest output energy by scanning all possible directions in the field. An energy distribution curve of the scanning field can be drawn by scanning all directions in the field and calculating the corresponding output energy of the sensor array. When the scanning direction (θ,ϕ) is restricted to the leak hole, the output energy of the sensor array reaches a maximum and hence a peak point in the energy distribution curve. Because a one-dimensional linear array was used in the experiments, the pitch angle ϕ was not considered.

## 3. Experiment and Results

### 3.1. Experiments

**Figure 1:**
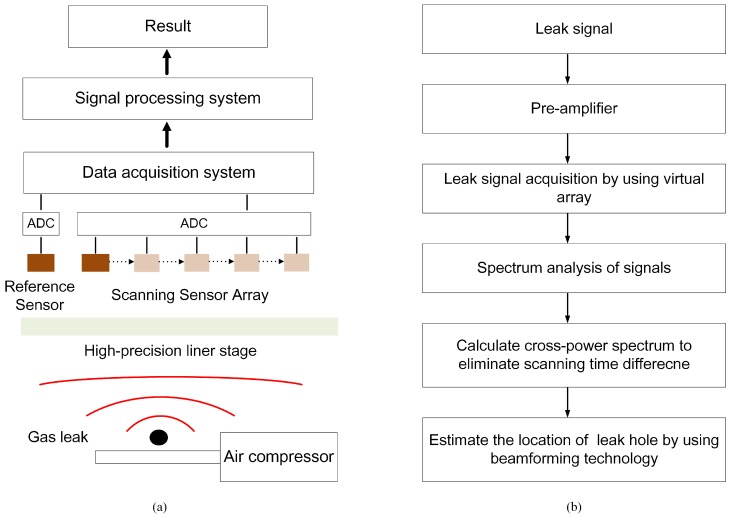
(**a**) Block diagram of experimental apparatus. (**b**) Flowchart for the process of estimating the location of the leak hole.

**Figure 2:**
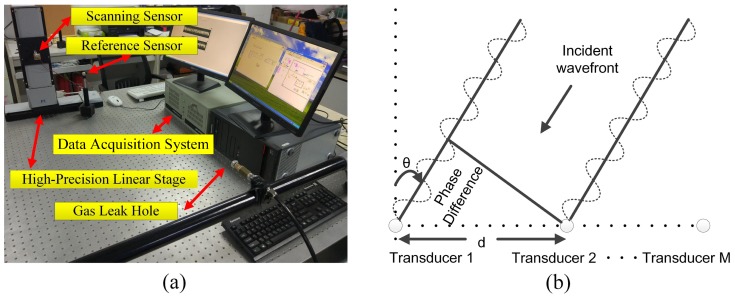
(**a**) Experimental apparatus. (**b**) Schematic diagram of the linear array receiving a signal.

To evaluate the effectiveness and operability of the proposed method, experiments were carried out on an optical platform with a size of 150 × 150 × 200 cm. The experimental apparatus block diagram and flowchart for estimating the location of the leak are shown in Figure 1, in which the ADC is analog-to-digital converter. Figure 1a illustrates two sensors being used to receive ultrasonic signals. One of the sensors (the reference sensor) is used to receive the reference signal at a fixed position and the other sensor (the scanning sensor) moves in intervals of 3 mm on a high-precision linear translation stage ((M-521.DG1, Physik Instrumente Corporation, Karlsruhe, Germany) with a range of 204 mm. Acoustic data from the sensor array were acquired by a fully digital dual-channel recorder (PCI 8552, ART Technology Corporation, Beijing, China) at a sampling rate of 1 MHz and sent to a PC. The saved experimental data were processed with MATLAB software. An air compressor with a nozzle provided the loading and thus the leakage pressure. The air compressor (QTS-1500X2, Outstanding Corporation, Taizhou, China) caused the ejection of air from the nozzle to simulate a leakage source. A continuous gas leak was simulated at a pressure of 7 bar from a circular shaped hole with a 0.5 mm diameter and the distance from the leak hole to the scanning array was 70 cm. The actual experimental setup and sensor arrangement are shown in Figure 2a.

The leakage acoustic signals are mainly distributed in the frequency range of 10–100 kHz and the largest energy difference is between a 40 kHz ultrasonic signal and the environmental noise [30]; thus, the localization results are calculated using signals at a frequency of 40 kHz. To ensure an optimal response, FUS-40CR piezoelectric ultrasonic sensors (Fuji Ceramics Corporation, Shizuoka-ken, Japan) were selected to collect the leakage signals. The sensor has a fixed working frequency of 40 kHz with an average sensitivity of −46 dB. The key technical specifications of the ultrasonic sensors are summarized in Table 1.

### 3.2. Characteristics of Acoustic Emission Leakage Signals

The beamforming algorithm estimates the location of a leakage source using the spatial characteristics of the sound field. Here, the spatial characteristics of the sound field refer to the phase change in the signal due to spacing. Therefore, we first verified whether the sound field is time-stationary, that is, whether the phase change in the signal is time-invariant. In a time-stationary sound field, the phase difference between different array elements is only related to the spatial position. Figure 2b is a schematic diagram of the line array receiving signals; the incident direction of a plane wave is θ and the spacing between two adjacent elements is *d*. The phase difference between the signals received by the *i*th array element and the first array element can be expressed as

(19) $$\Delta \varphi_{i}=2\pi f\frac{(i-1)dsin\theta }{c}.$$

Equation (19) shows that the phase difference is only related to the spacing between adjacent sensors and linearly increases as the spacing increases. The actual phase difference between the array element signals is calculated by Equation (9). If a linear phase change is obtained that is the same as the theoretical calculation, it can be proved that the leakage signals received by the array belong to a time-stationary sound field. Because of the characteristics of the phase angle calculation function, the actual phase difference calculated by Equation (9) ranges from 0 to 2π. The phase difference is unwrapped to facilitate the observation of the change characteristics of the phase difference.

**Figure 3:**
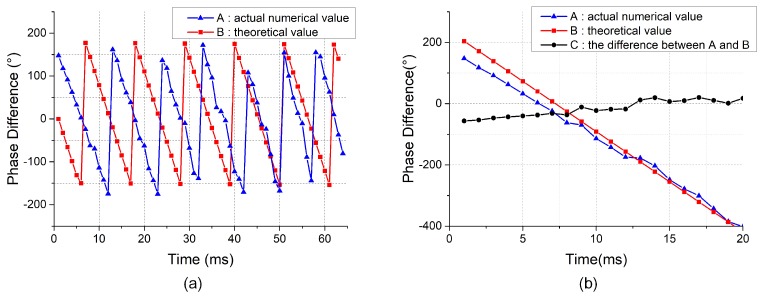
(**a**) Theoretical value and actual numerical value of the phase difference between the scanning signal and the reference signal. (**b**) Phase difference after unwrapping.

Figure 3a shows the theoretical value and actual numerical value of the phase difference between array elements. In Figure 3a, the blue line represents the actual numerical value and the red line represents the theoretical value. Table 2 shows the phase difference after unwrapping and Figure 3b is the phase difference curve after unwrapping. As can be seen from Figure 3a,b, the actual numerical value and the theoretical value have the same change law. Since the spacing between virtual elements is the same, the phase difference increases or decreases linearly. The magnitude of the phase difference change depends on the direction of the ultrasound signal. The slopes of the red and blue lines are −32.0 and −32.8, respectively. Therefore, it can be proved that the leakage signals received by the array belong to a time-stationary sound field.

### 3.3. Interference Analysis of the Algorithm

In the time domain, 40 kHz leakage signals are used to estimate the location of the leak source and other frequency signals are filtered out. Therefore, the background noise does not interfere with the calculation.

**Figure 4:**
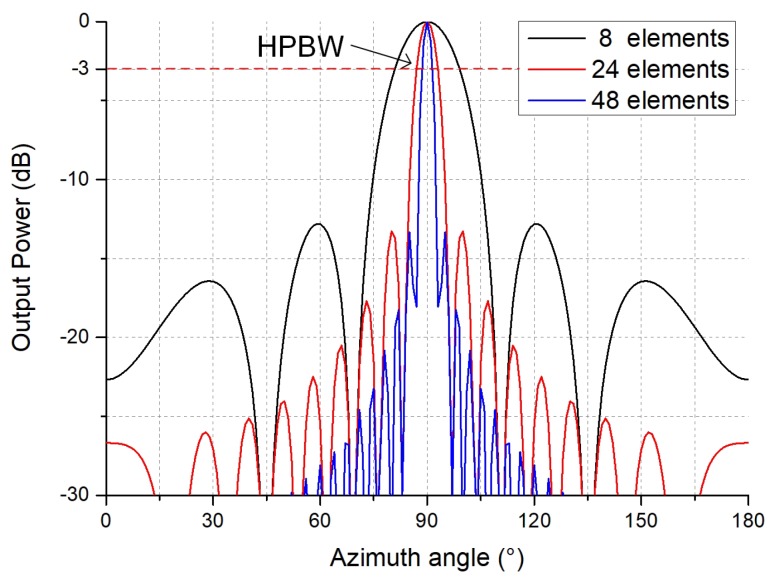
Beam pattern of arrays with different numbers of elements. HPBW: half power beam widths.

In the spatial domain, beamforming technology is used to estimate the location of the leak source. We present the beam pattern of an 8-element array, a 24-element array and a 48-element array. The beam patterns are shown in Figure 4, which shows that when the number of array element is 8, 24 and 48, the half power beam widths (HPBW) of the three arrays are 19.1${}^{{}^{\circ}}$, 6.5${}^{{}^{\circ}}$ and 2.5${}^{{}^{\circ}}$ respectively. Moreover, the number of side lobes and the amplitude of each side lobe both decrease. It can be seen from Figure 4 that the directivity of array increases as the array element increases. Taking an 8-element array as an example, the sensor array accepts a larger angle range of signals near the desired angle and signals at other angles interfere with the estimated leakage position. In our method, an array of 48 elements is used and its beam pattern has a narrow main lobe width. Therefore, the SNR of the signal and the orientation accuracy increase with increasing array element.

### 3.4. Results and Discussion

**Figure 5:**
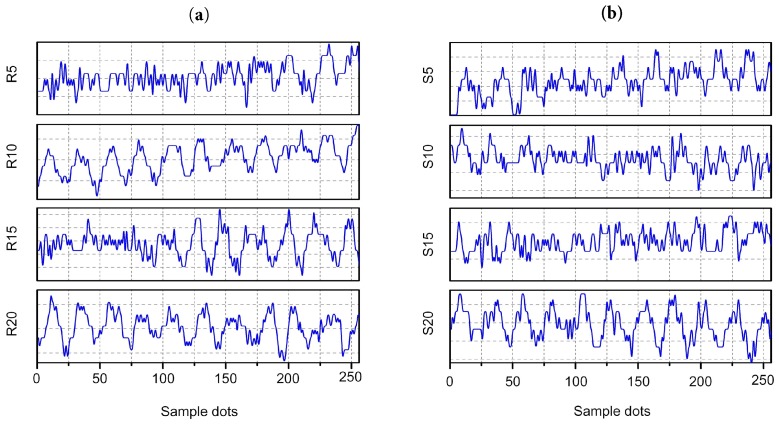
(**a**) The signals received by the reference sensor. (**b**) The signals received by the scanning sensor.

**Figure 6:**
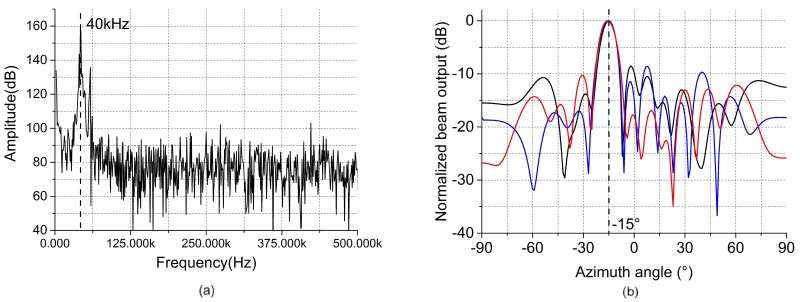
(**a**) The frequency spectrum of the leakage signal. (**b**) Localization results under identical experimental conditions.

The effect of the virtual ultrasonic sensor array technology on the actual gas leakage location is discussed in this section. In the experiment, the air outlet of the air pump is the source of the leakage. Figure 5a,b show the leakage signals received by the reference sensor and scanning sensor, respectively. Figure 6a shows the frequency spectrum of the leakage signal. As can be seen from Figure 6a, the leakage signal energy is mainly concentrated at the 40 kHz signal. Three experiments were carried out under identical experimental conditions to verify the stability of the algorithm. A virtual array of 48 elements was used and the array was 80 cm away from the leakage source. The leak hole was placed in a −15.0° position relative to the array. As reported in Figure 6b, the three sets of localization results are −15.2°, −15.6° and −16.1°, respectively. These experimental results indicate that this method can stably and accurately locate leakage sources.

**Figure 7:**
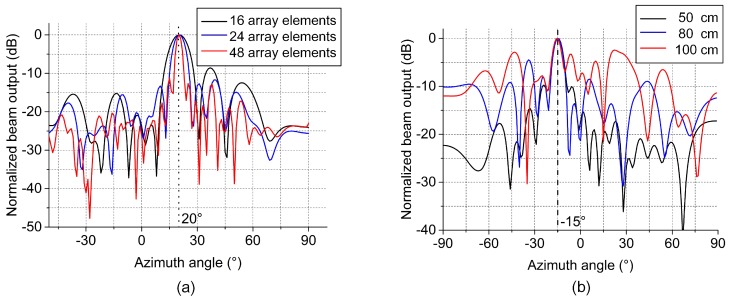
(**a**) The results of different numbers of array elements. (**b**) The results of different distances.

In order to study the influence of the sensor number on the localization performance of the algorithm, we studied the localization results of three separate linear arrays with 16, 24 and 48 sensors. In the three sets of experiments, only the number of sensors differed and all other experimental parameters were the same. The localization results are shown in Figure 7a, in which the black line is the localization result of 16 sensors and the blue and red lines are the localization results of 24 and 48 sensors, respectively. Figure 7a shows that the direction of the leakage source is 20.0°. It can be seen from Figure 7a that when the number of sensors is 16, 24 and 48, the main lobe widths in the localization results are 9.5°, 6.0° and 2.5°, respectively. The maximum side lobes are −8.5 dB, −11.72 dB and −11.17 dB, respectively. The results show that as the array element number increases, the width of the main lobe becomes narrower and the maximum side lobe becomes lower. A narrower main lobe indicates a better directivity of the array and a lower side lobe enhances the anti-interference ability.

In the following, we describe the effect of the distance between the leakage source and the virtual array on the localization algorithm performance. In three sets of experiments, we placed the leakage source at a distance of 50, 80 and 100 cm from the virtual array and all other experimental parameters were the same. The results are shown in Figure 7b, which shows that when the leakage source is 50, 80 and 100 cm from the array, the maximum side lobes are −9.7, −4.4 and −2.4 dB. Moreover, the number of side lobes and the amplitude of each side lobe both increase. As the distance increases, the attenuation of the signal also increases. Therefore, the SNR of the signal and the orientation accuracy are reduced with increasing distance.

**Figure 8:**
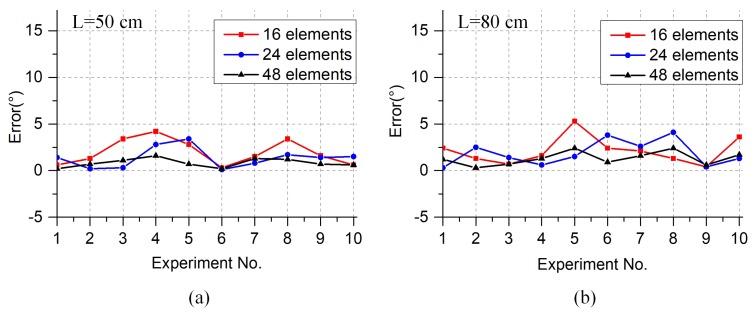
(**a**) Error analysis when the distance is 50 cm. (**b**) Error analysis when the distance is 80 cm.

A series of experiments were conducted to verify the accuracy of the method. In order to eliminate the influence of the direction of the leakage source on the experimental results, we ensured that the direction angle was $-15^{{}^{\circ}}$ in each test. Using the same measurement method with different numbers of array elements and different distances, the localization error was analyzed. Ten scenarios were tested and the localization errors are shown in Table 3 and Figure 8. The localization errors are defined as the angle between the estimated location and the actual location. We cannot derive useful rules from individual experimental scenarios. However, when the mean error and the standard deviation are studied and the distance is constant, we can see that as the number of elements increases, the localization error decreases. We can also conclude that when the number of array elements is constant, the localization error increases as the distance increases.

The localization errors of the 10 scenarios are also shown in Figure 8, from which we can draw the same conclusion. The experimental results show that when the number of array elements is 48 and the distance between the leak hole and sensor array is 50 cm, the average error of gas leakage localization is only 0.8°.

## 4. Conclusions

The signals received by the virtual ultrasonic sensor array are proved to belong to a time-stationary sound field, so the virtual beamforming algorithm can be used to estimate the direction of the leakage source. Experiments were carried to evaluate the effectiveness and operability of the proposed method. In the experiments, we used only two sensors to form a large-aperture sensor array of 48 elements. The experimental results show that this method can improve the localization resolution and the stability performance of the algorithm is good. The impacts of various factors on the performance of the localization technique are compared and discussed, including the number of sensors and the distance between the leak hole and the array. The results suggest that a sufficient number of array elements should be used to ensure that the localization resolution is within an appropriate detection range. Furthermore, we carried out several experiments to verify the accuracy of the method by varying the sensor number and the distance between the leak hole and virtual array. We draw the same conclusions from the results of these experiments (Figure 8), which show that when the virtual array has 48 elements and the distance between leak hole and sensor array is 50 cm, the average error of gas leakage localization is only 0.8°. In summary, the proposed gas leakage detection method based on a virtual ultrasonic sensor array shows high potential for locating gas leakage sources with high resolution.

For practical applications, traditional sensor arrays have the advantage of rapid measurement. But the localization resolution of these methods with few sensors is limited because of insufficient spatial sampling. In order to obtain high-resolution gas leakage localization results, a 48-element VPA of only two sensors is used in this paper. Compared with conventional methods, the VPA-based approach can not only improve the resolution of gas leakage localization but also avoid array calibration issues. Furthermore, the use of scanning array resolves several disadvantages of traditional arrays, such as high cost, poor flexibility and system complexity. But the scanning time is a crucial factor for practical application. For example, the scanning time of our method with 48 array elements is 20 s. In the future, it is a challenge to design and improve the efficient algorithm for reconstructing acoustic field of gas leakage by a few spatial sampling, that is, shorter scanning time. Scene reconstruction with the sparsity of leakage sources make possible for application of compressed sensing to above-mentioned issue.

## Figures and Tables

**Table 1:** Sensor main technical features.

Item	Value
Diameter	15 mm
Directivity	50 deg
Sensitivity	−16 dB
Center frequency	40 kHz
Detection distance	0.2–6 m

**Table 2:** Phase difference after unwrapping.

Time (ms)	A: Actual Numerical Value (${}^{{}^{\circ}}$)	B: Theoretical Value (${}^{{}^{\circ}}$)	C: Difference between A and B (${}^{{}^{\circ}}$)
2	117.79	171.31	−53.51
4	62.52	105.69	−43.17
6	2.94	40.07	−37.14
8	−62.17	−25.54	−36.62
10	−113.79	−91.16	−22.63
12	−174.48	−156.78	−17.70
14	−203.07	−222.39	19.32
16	−278.19	−288.01	9.82
18	−342.82	−353.62	10.81
20	−401.74	−419.24	17.50

**Table 3:** Localization error with different numbers of array elements and different distances.

Number	Error (${}^{{}^{\circ}}$) (L = 50 cm)			Error (${}^{{}^{\circ}}$) (L = 80 cm)		
	16 Elements	24 Elements	48 Elements	16 Elements	24 Elements	48 Elements
1	0.6	1.4	0.2	2.4	0.3	1.2
2	1.3	0.2	0.7	1.3	2.5	0.3
3	3.4	0.3	1.1	0.7	1.4	0.7
4	4.2	2.8	1.6	1.6	0.6	1.3
5	2.8	3.4	0.7	5.3	1.5	2.4
6	0.3	0.1	0.2	2.4	3.8	0.9
7	1.5	0.8	1.3	2.1	2.6	1.6
8	3.4	1.7	1.2	1.3	4.1	2.4
9	1.6	1.4	0.7	0.4	0.4	0.6
10	0.6	1.5	0.6	3.6	1.3	1.7
Mean error	2.0	1.4	0.8	2.1	1.9	1.3
Standard deviation	1.3	1.0	0.4	1.3	1.3	0.7

## References

[1] Aamo O. (2016). Leak Detection, Size Estimation and Localization in Pipe Flows. IEEE Trans. Autom. Control.

[2] Sampaolo A., Patimisco P., Giglio M., Chieco L., Scamarcio G., Tittel F.K., Spagnolo V. (2016). Highly sensitive gas leak detector based on a quartz-enhanced photoacoustic SF6 sensor. Opt. Express.

[3] Sun A.Y., Lu J., Freifeld B.M., Hovorka S.D., Islam A. (2016). Using pulse testing for leakage detection in carbon storage reservoirs: A field demonstration. Int. J. Greenh. Gas Control.

[4] Murvay P.S., Silea I. (2012). A survey on gas leak detection and localization techniques. J. Loss Prev. Process Ind..

[5] Mostafapour A., Davoudi S. (2013). Analysis of leakage in high pressure pipe using acoustic emission method. Appl. Acoust..

[6] Miller R., McIntire P. (1987). Acoustic emission testing. American Society for Nondestructive Testing. Nondestruct. Test. Handb..

[7] Chou H., Mouritz A., Bannister M., Bunsell A.R. (2015). Acoustic emission analysis of composite pressure vessels under constant and cyclic pressure. Compos. Part A Appl. Sci. Manuf..

[8] Liao P., Cai M. (2013). High accuracy method of ultrasonic gas leak direction detection based on time delay estimation. J. Beijing Univ. Aeronaut. Astronaut..

[9] Meng L., Jia Z., Yan S., Gao S. (2011). Location technology of mix of TDOA and Generalized Cross Correlation. Comput. Meas. Control.

[10] Kim M.S., Lee S.K. (2009). Detection of leak acoustic signal in buried gas pipe based on the time–frequency analysis. J. Loss Prev. Process Ind..

[11] Cui X., Yong Y., Ma Y., Lin M., Han X. (2016). Localization of CO_2_ leakage from transportation pipelines through low frequency acoustic emission detection. Sens. Actuators A Phys..

[12] Li S., Wen Y., Ping L., Jin Y., Dong X., Mu Y. (2014). Leak location in gas pipelines using cross-time–frequency spectrum of leakage-induced acoustic vibrations. J. Sound Vib..

[13] Xu C., Gong P., Xie J., Shi H., Chen G., Song G. (2016). An acoustic emission based multi-level approach to buried gas pipeline leakage localization. J. Loss Prev. Process Ind..

[14] Quy T.B., Muhammad S., Kim J.M. (2019). A Reliable Acoustic Emission Based Technique for the Detection of a Small Leak in a Pipeline System. Energies.

[15] Han X., Zhao S., Cui X., Yan Y. (2019). Localization of CO_2_ gas leakages through acoustic emission multi-sensor fusion based on wavelet-RBFN modeling. Meas. Sci. Technol..

[16] Tian H., Qiang P., Liu Y., Liu X., Hu D. (2012). Near-field beamforming analysis for acoustic emission source localization. Ultrasonics.

[17] Tao W., Wang D., Yu P., Wei F. (2015). Gas leak localization and detection method based on a multi-point ultrasonic sensor array with TDOA algorithm. Meas. Sci. Technol..

[18] Zhang Y., Wang J., Bian X., Huang X., Qi L. (2017). A continuous gas leakage localization method based on an improved beamforming algorithm. Measurement.

[19] Xu B., Yu Z., Yibo L., Xiaoyue G., Shijiu J. (2015). A new method of using sensor arrays for gas leakage location based on correlation of the time-space domain of continuous ultrasound. Sensors.

[20] Yan Y., Cui X., Guo M., Han X. (2016). Localization of a continuous CO_2_ leak from an isotropic flat-surface structure using acoustic emission detection and near-field beamforming techniques. Meas. Sci. Technol..

[21] Wang T., Wang X., Hong M. (2018). Gas Leak Location Detection Based on Data Fusion with Time Difference of Arrival and Energy Decay Using an Ultrasonic Sensor Array. Sensors.

[22] Yan Y., Shen Y., Cui X., Hu Y. (2018). Localization of multiple leak sources using acoustic emission sensors based on MUSIC algorithm and wavelet packet analysis. IEEE Sens. J..

[23] Comesana D.F., Holland K.R., Escribano D.G., de Bree H.E. (2014). An Introduction to Virtual Phased Arrays for Beamforming Applications. Arch. Acoust..

[24] Curlander J.C., McDonough R.N. (1991). Synthetic Aperture Radar- Systems and Signal Processing.

[25] Cutrona L.J. (1975). Comparison of sonar system performance achievable using synthetic-aperture techniques with the performance achievable by more conventional means. J. Acoust. Soc. Am..

[26] Holl P.M., Reinhard F. (2017). Holography of Wi-fi radiation. Phys. Rev. Lett..

[27] Luo Z.W., Comesana D.F., Zheng C.J., Bi C.X. (2019). Near-field acoustic holography with three-dimensional scanning measurements. J. Sound Vib..

[28] Clement G., White J., Hynynen K. (2000). Investigation of a large-area phased array for focused ultrasound surgery through the skull. Phys. Med. Biol..

[29] Dayou M.A. (1988). Development of The Law of Turbulent Jet Noise. Acta Acust..

[30] Cui X., Yan Y., Guo M., Han X., Hu Y. (2016). Localization of CO_2_ Leakage from a Circular Hole on a Flat-Surface Structure Using a Circular Acoustic Emission Sensor Array. Sensors.

